# Treatment hurts: Lay theories of graded exposure in the treatment of four anxiety disorders

**DOI:** 10.1080/13642537.2013.810657

**Published:** 2013-07-11

**Authors:** Adrian Furnham, Emma Wilson, Amy Chapman, Raj Persuad

**Affiliations:** Research Department of Clinical, Educational and Health Psychology, University College London, London, UK

**Keywords:** lay theories, psychotherapy, graded exposure, anxiety disorders, affect, théories profanes, psychothérapie, exposition graduelle, troubles anxieux, affect

## Abstract

**Objective:**

This paper concerned the perceived suffering/side effects caused by various well-known treatments for personal problems. It looked at whether people understood whether potentially painful treatments that confront negative aversive affect were effective or not.

**Method:**

In total, 106 participants completed a long questionnaire assessing the ‘psychological pain’ ratings of 30 psychotherapy treatments, varying in fear exposure, for four relatively common anxiety disorders: social phobia, agoraphobia, post-traumatic stress disorder, and obsessive compulsive disorder.

**Results:**

Factor analytic results revealed four clear factors underlying lay efficacy beliefs of psychotherapy interventions, varying in fear exposure: talking therapies, fear confrontation, fear avoidance, and alternative therapies. Talking therapies were rated the most effective across all disorders, but also the most painful. Fear avoidance therapies were rated the least effective and, along with alternative medicine, the least painful. Treatments involving fear exposure were rated the most painful. Regression analysis revealed talking therapies to be rated more efficacious by younger subjects than older subjects.

**Conclusion:**

Most people seem able to differentiate between the efficacies of interventions for different anxiety disorders and hold consensually held optimistic conceptions about the usefulness of psychotherapy treatments and counseling that involve fear exposure, despite knowledge of the psychophysical side effects that these therapies often entail. They favored talking cures over others, but that may have been due to misleading items in the questionnaire.

## Abstract (ES)

**Objetivo:**

Este artículo explora el sufrimiento o los efectos secundarios percibidos como causados por la influencia de varios bien conocidos tratamientos para dificultades emocionales.

**Método:**

106 pacientes en total completaron un largo cuestionario para evaluar el alcance del dolor psicológico de 30 tratamientos terapéuticos variando en exposición al miedo, para cuatro trastornos de ansiedad relativamente comunes: fobia social, agorafobia, trastorno postraumático estresante y trastorno obsesivo-compulsivo.

**Resultados:**

Los resultados de análisis de factores revelaron claramente cuatro factores subyacentes en las creencias laicas acerca de las intervenciones terapéuticas variando en exposición al miedo: las terapias de conversación, fueron consideradas como las más efectivas en todos los trastornos pero también las más dolorosas; las terapias de evitación del miedo, las menos efectivas y junto con medicina alternativa, las menos dolorosas; los tratamientos que implicaron exposición al miedo fueron considerados los más dolorosos. El análisis de la regresión reveló que los sujetos más jóvenes en contraposición a los más viejos, consideraron las terapias de conversación como las más eficaces.

**Conclusión:**

La mayoría de la gente parece capaz de diferenciar la eficacia de las intervenciones para diferentes trastornos de ansiedad, y existe un consenso que sostiene concepciones optimistas acerca de la utilidad de los tratamientos terapéuticos y de orientación psicológica que implican exposición al miedo; a pesar de los efectos psicológicos secundarios que estas terapias acarrean, favorecen las terapias de conversación sobre las otras, pero ésto puede deberse a algunos puntos engañosos en el cuestionario.

## Abstract (IT)

**Obiettivo:**

Questo studio riguarda gli effetti collaterali percepiti di sofferenza causati da vari noti trattamenti per problemi personali. Si è preso in considerazione se le persone avessero compreso se il trattamento potenzialmente doloroso che affronta effetti negativi deterrenti, fosse efficace o meno.

**Metodo:**

In totale i 106 partecipanti hanno compilato un lungo questionario per valutare l'indice di “sofferenza psicologica” di 30 trattamenti di psicoterapia, con variazioni di esposizioni alla paura, per quattro disturbi d'ansia relativamente comuni: sociofobia, agorafobia, disturbo post-traumatico da stress (PTSD) e disturbo ossessivo compulsivo (OCD).

**Risultato:**

I risultati degli elementi dell'analisi rivelano quattro chiari elementi che supportano la convinzione profana nell'efficacia degli interventi di psicoterapia, di esposizione variabile alla paura: le terapie della parola, l'affrontare la paura, l'evitare la paura e le terapie alternative. Le terapie della parola sono state valutate le più efficaci tra tutti i disturbi, ma anche le più dolorose. Le terapie per evitare la paura sono state valutate le meno efficaci, insieme alle medicine alternative, e le meno dolorose. I trattamenti che comprendono l'esposizione alla paura sono stati valutati i più dolorosi. Un'analisi regressiva ha rivelato che le terapie della parola sono state valutate più efficaci dai soggetti più giovani che da quelli meno giovani.

**Conclusione:**

Molte persone sembrano in grado di distinguere tra l'efficacia degli interventi per disordini d'ansia differenti e mantengono consensualmente percezioni ottimiste nei confronti dell'utilità dei trattamenti di psicoterapia e counselling che implicano l'esposizione alla paura, nonostante la conoscenza degli effetti collaterali psico fisici che queste terapie spesso comportano. Essi hanno preferito la terapia della parola sulle altre, ma ciò può essere stato dovuto a degli elementi fuorvianti nel questionario.

## Abstract (FR)

**Objectif:**

Le sujet de cet article est la perception de la souffrance/des effets secondaires causés par différents traitements connus des problèmes personnels. Il cherche à savoir si les gens comprennent en quoi des traitements potentiellement pénibles confrontant des affects négatifs d'aversion peuvent être efficaces ou pas.

**Méthode:**

Au total 106 participants ont rempli un long questionnaire évaluant ‘la souffrance psychique’ de trente traitements psychothérapeutiques, variant dans leur niveau d'exposition à la peur, pour quatre troubles anxieux communs: la phobie sociale, l'agoraphobie, le stress post-traumatique (PTSD) et les troubles obsessionnels compulsifs (OCD).

**Résultats:**

Les résultats du facteur analytique ont révélés quatre facteurs distincts sous-tendant les croyances profanes en ce qui concerne l'efficacité des interventions psychothérapeutiques variant dans leur degré d'exposition à la peur. Les thérapies par la parole ont été notées comme étant les plus efficaces pour tous les troubles mais également les plus douloureuses. Les thérapies basées sur l’évitement de la peur ainsi que la médecine alternative ont été notées comme étant les moins efficaces mais aussi les moins douloureuses. Les traitements impliquant une exposition à la peur ont été notées comme étant les plus douloureuses. L'analyse régressive a révélé que les thérapies par la parole ont été notées comme étant plus efficaces plus par des sujets jeunes que par des sujets plus âgés.

**Conclusion:**

La plupart des gens semblent capables de différencier les efficacités des interventions pour différents troubles anxieux et ont des conceptions dans l'ensemble optimistes sur l'utilité des traitements psychothérapeutiques qui impliquent l'exposition à la peur bien qu'ils soient conscients des effets psychophysiques souvent associés à ces thérapies. Ils préfèrent les thérapies par la parole plutôt que d'autres mais ceci est peut-être dû à la manière dont les questions étaient formulées.

## Introduction

There is a growing body of research into lay theories of mental disorders, their causes and consequences (Angermeyer & Matschinger, 1996; Angermeyer, Matschinger, & Holzinger, 1998; Dammann, 1997; Furnham, 1988; Furnham, Wardley, & Lillie, 1992; Furnham, Pereira, & Rawles, 2001; Ojanen, 1992; Oyefeso, 1994; Pistrang & Barker, 1992; Shapiro, 1995). This study is concerned with lay people's beliefs (as potential clients) about the efficacy and psychological pain/discomfort resulting from using a variety of psychotherapy interventions in the treatment of four relatively common anxiety disorders. It is concerned with the potentially mistaken notions lay people have about the experience and effectiveness of psychotherapy, particularly concerning managing affect.

### Lay theories and mental health literacy

The term ‘mental health literacy’ is defined as ‘knowledge and beliefs about mental disorders which aid their recognition, management or prevention’ (Jorm et al., 1997a, 1997b; Jorm et al., 1997). Jorm and colleagues have contributed substantial research evidence in this area (Chen, Parker, Kua, Jorm, & Loh, 2000; Jorm et al., 2000) and interest has gathered pace over recent years (Goldrey, Fisher, & Wilson, 2001; James et al., 2002; Mubbashar & Farooq, 2001). Studies have been conducted on the public's ‘mental health literacy’ of specific disorders including schizophrenia and depression (Brewin & Furnham, 1986; Jorm et al., 1997a, 1997b), and neuroticism (Furnham, 1984). Lay people also appear more confident in specifying the ‘cure’ for problems rather than their perceived cause (Furnham & Henderson, 1983) and the majority believe that mental disorders are treatable (McKeen & Corrick, 1991; Reiger et al., 1988) but that some psychiatric treatments are considered generally unhelpful (Reiger et al., 1988; Sims, 1993). This is in spite of the majority being unable to correctly identify mental disorders from short written vignettes (Jorm et al., 1997a), as well as specify either their causes or the most efficacious treatments.

### Lay perceptions of psychotherapy

Public perceptions of psychotherapists and the process of psychotherapy have been speculated to have important implications in terms of the number and type of individuals who choose to seek psychological treatment (Furnham & Wardley, 1990; Halgin, Weaver, & Donaldson, 1985; Wong, 1994). In addition to these influences on potential clients and on their actual experience of treatment, popular perceptions of psychotherapy are likely to have significant implications for public policy and mental health reform (e.g. Knapp & Kamin, 1993; Pallak & Kilburg, 1986). Therefore, obtaining a more thorough understanding of the nature of popular perceptions and their antecedents may be helpful in designing interventions to modify negative attitudes towards seeking help (Fischer & Turner, 1970).

The general public (as potential clients) is increasingly faced with a bewildering array of psychotherapy interventions available, although some are clearly similar in theory and practice. These include seeing a therapist, attending training courses or focus groups, observation and/or taking medication, or getting hypnosis. Deciding whether or not to seek help is associated with a range of factors including the availability of services, financial costs, and individual sociodemographic and psychological variables (Sheikh & Furnham, 2000). It is also crucially associated with the perceived effort required in, and possible psychological pain associated with, treatment which is the focus of this paper. The term psychological pain refers here to the distress associated with the treatment process.

The groups least likely to utilize mental health services are men, older people, and people from ethnic minorities who are more likely to display avoidance behavior, resistance to treatment, and denial of mental illness (Leong & Zachar, 1999). Aside from these influential factors, two major criteria that lay people factor into their choice or recommendation of a therapy presumably is the perceived efficacy of the treatment and the associated side effects for specific psychological issues. For example, counseling is frequently considered most helpful (McKeen & Corrick, 1991) and expectations of counseling involve talking to an experienced expert who can be trusted (Tinsley & Harris, 1976). Prospective patients of many of the talking therapies, particularly psychoanalytic therapies, often seem ignorant of the psychic effort that they are required to make and the possible emotional pain that results from their therapy (Furnham, 2010). It is expectations such as these that facilitate or hinder the effectiveness of therapy (Apfelbaum, 1958) as well as the choice of therapy.

Various studies have addressed lay beliefs about the best cure for, and ways of overcoming, psychological problems (Knapp & Karabenick, 1985). Together, they replicated the factor structure and cure-specific perceptions of the efficacy of different cures (Furnham & Henley, 1988; Henley & Furnham, 1988) and addictions (Furnham & McDermott, 1994), emphasizing the importance of self-control and, to a lesser extent, professional help, depending on the nature of the disorder. In a series of three studies, Furnham and Wardley (990, 1991, 1992) investigated lay people's theories regarding the efficacy of various psychotherapy interventions and the prognosis of different disorders. They identified an interpretable underlying factor structure, with lay people discriminating quite clearly between the efficacy of 22 different therapies. It was further found that subjects felt largely optimistic about the influence of psychotherapy on various psychological problems and participant age and education were significant predictors of these beliefs.

One factor that was predictably related to lay theories about psychotherapy was participant's direct or indirect knowledge (through reading) of psychological ideas and therapies. The more experienced subjects had, the more skeptical they were about the usefulness of various treatments. Furnham, Wardley, and Lillie (1992) found, when compared to lay adults, psychotherapists, and students were more skeptical and pessimistic about the efficacy of therapy and prognosis for many illnesses. Knowledge about psychological cures led to a greater awareness of the limited benefits of therapy. However, this finding was not replicated by Furnham (2010) in his investigation of lay attitudes towards and understanding of psychotherapy in treating two psychotic (bipolar and schizophrenia) and two neurotic (depression and obsessive compulsive) disorders. It was confirmed, however, that participants were generally positive about the experience of psychotherapy but were curiously naïve about the efficacy of psychotherapy.

### Therapy options

Psychotherapies involving cognitive, affective, and behavioral procedures have been established as empirically supported treatments for anxiety disorders (e.g. Chambless & Ollendick, 2001). Most cognitive and behavioral techniques are derived from theoretically coherent and empirically validated models of anxiety disorders and provide a consistent relationship between the treatment techniques and symptoms. In addition, from the client's perspective, they have fewer (at least biological and pharmacological) undesirable side effects and no addictive potential that may occur with other therapies, including medication.

For many anxiety disorders, particular situations are avoided because of the aversive affect they produce: this is judged by themselves and professionals as unmanageable and needs treatment. All therapies accept the idea that affect needs to be faced (through behavioral exposure or talking about past events) and that in doing so it becomes much more manageable. Developments in radical behaviorism are particularly strong on this idea (Hayes, Strosahl, & Wilson, 1999; Linehan, 1993). Exposure to affect has also always been central to the psychodynamic and humanistic therapies (Bornstein, 1989).

The most widely employed behavioral technique to treat anxiety disorders is graded exposure therapy – confrontation with the feared situation in real life (*in vivo*), in imagination or in role playing situations during therapy. Specifically, it involves a hierarchy of progressively more challenging confrontations with the phobic stimulus. It often begins with a mildly difficult activity with a sequential series of steps that eventually culminate with an activity that would be challenging for most people (Marks & O'Sullivan, 1988). Research has found there to be no additive effect of combining cognitive techniques for anxiety disorders (Deacon & Abramowitz, 2004) while other studies have indicated that talking cures are thought to be efficacious for neurotic disorders. Exposure therapy has been found to reduce agoraphobia, panic, compulsive rituals, work, and social disabilities, however, despite its success, treatment choices are frequently made according to patient preference. The associated anxiety and discomfort that can arise from fear exposure can, in the short-term, result in clients not agreeing to this therapy.

### This study

The present study aims to extend the catalogue of lay theories about the treatment of psychological problems, previously identified as being organized, multifaceted, and interconnected. Specifically, it is concerned with the investigation of efficacy beliefs of various psychological treatments, varying in systematic exposure, for four relatively common anxiety disorders about which there is relatively scant research: social phobia, agoraphobia, post-traumatic stress disorder (PTSD), and obsessive compulsive disorder (OCD). It is likely that individuals who know or have known people affected by one or more of these disorders will have formulated opinions regarding their cause and treatment.

Considering the research findings of previous efforts, it is predicted that psychotherapy interventions requiring fear exposure, either in real time or through imagination with a therapist, will be considered the most effective treatments but also the most painful (H^1^). Therapy interventions that involve minimal or no fear exposure will receive the lowest efficacy ratings *and* be considered the least painful (H^2^).

It is further predicted, in line with previous lay theories research, that there will be a significant effect of ‘knowledge or experience of psychotherapy’ Specifically, it is hypothesized that participants who are more knowledgeable (in education and experience) will have more realistic expectations of each of the interventions and be more skeptical about the efficacy of different treatments for each of the disorders than those who are less knowledgeable (H^3^).

## Method

### Participants

In all, 106 participants participated in this study, 72 of whom were female. Their age ranged from 16 to 61 with a mean age of 25.00 years (SD = 8.28). The majority were of European Caucasian descent (62.3%), however other ethnic groups were also represented, including Asian (22.6%) and Afro-Caribbean (1.9%). In terms of marital status, 59.4% of participants were single and 38.6% were either in a relationship or married. Over half of participants self-reported no religious convictions (57.5%) and 0.9% were very religious, while only 9.5% of participants self-reported strongly held left- or right-wing political affiliations. Finally, in terms of educational attainment, 48.1% had studied to undergraduate level and 16.1% had continued with postgraduate studies.

When asked if they had ever been clinically diagnosed with a mental disorder, the majority (88.7%) responded No. In all, 39.6% of participants had formally studied a clinical health-related subject (i.e. psychology, psychiatry, and medicine) while 44.3% reported no formal study of these disciplines. Finally, when asked to estimate their own knowledge of psychiatric disorders, 39.9% reported very little or no knowledge and 7.5% reported very good understanding.

### Measures

Participants completed a 120-item questionnaire, which contained questions regarding treatment options for four anxiety disorders as classified by DSM: social phobia, agoraphobia, PTSD, and OCD. This was a new measure derived for this study. A brief description of each disorder, based on the DSM IV typology, was presented at the top of the 30 treatment options. Participants were asked to consider the efficacy of each treatment option with regard to that specific disorder. Specifically, for each treatment option, participants were required to rate on a seven-point Likert scale: (a) How well does it work? and (b) How painful is it? Responses were anchored by: (1) Very well; Very painful and (7) *Not at all well; Not at all painful*. They were also asked how long the treatment takes and how much it costs but this data was not analyzed in this paper. The ‘treatments’ were derived from various sources including interviews with lay people about what they thought would best help/treat these problems. The aim was to be comprehensive and it was decided not to use technical terms like CBT, psychodynamic theory, or client-centered counseling and use lay language that is better understood. Pilot work ensured that the questionnaire was fully understandable to most people.

### Procedure

Department ethics permission was sought and received. Participants were approached in a number of public settings including libraries, coffee bars, and railway stations in the Greater London area. Approximately one-third of those approached refused their participation on the basis that they were too busy. Completion of the questionnaire took approximately twenty-five to forty minutes. Participants were asked to provide demography, including age, and educational attainment, as well as information regarding their previous knowledge of mental illness and psychotherapy: Have you ever been diagnosed with a mental disorder? (Yes, No, Not sure, and Prefer not to say); Have you ever formally studied any of the following subjects at degree level or higher? (Psychology, Psychiatry, Medicine, Psychoanalysis, and Child Care): On a scale of 1 (not at all knowledgeable) to 7 (very knowledgeable), how would you rate your knowledge of psychiatric disorders? Following successful completion of the questionnaire, participants were thanked and debriefed where possible.

## Results

### Experience of psychology

Preliminary inspection of the Experience of Psychology responses revealed that they were intercorrelated. Specifically, the correlations between Clinical-related subjects studied to degree level and knowledge of psychiatric disorders reached significance (*r* = −.50, *p* < 0.001). This result suggests that participants who had studied Psychology, Psychiatry, or Medicine to degree level were more likely to self-report as more knowledgeable of psychiatric disorders than those who had had not.

### Efficacy of psychotherapy treatments

Table 1 shows the mean responses to each item (1 = very effective, 7 = Not at all effective). Items that elicited the strongest efficacy beliefs in treating social phobia, agoraphobia, PTSD, and OCD showed a similar pattern across disorders, all of which involved seeing a therapist: One who requires you to face your fears (item 27: social phobia: *X = 2.72*; agoraphobia: *X* = 2.87; OCD: 2.83); one who listens attentively (item 28: social phobia: *X* = 2.91; agoraphobia: *X* = 2.91; PTSD: *X* = 2.90; OCD: *X* = 3.01); one who the patient enjoys talking to (item 29: social phobia: *X* = 2.93; PTSD: *X* = 3.07; OCD: *X* = 3.04); and one who is supportive and sympathetic (item 26: social phobia: *X* = 3.04; agoraphobia: *X* = 3.11; PTSD: *X* = 2.94; OCD: *X* = 3.19).

**Table 1.** Means, standard deviations, and factor loadings for efficacy ratings of psychotherapy treatments.

			Anxiety	Disorder			Factor	Loading	
		Social				1	2	3	4
	Treatment	phobia	Agoraphobia	PTSD	OCD	(29.23%)	(13.99%)	(8.98%)	(5.11%)
26	Seeing a supportive and sympathetic therapist	3.04 (1.37)	3.11 (1.30)	2.94 (1.26)	3.19 (1.32)	.83			
28	Seeing a therapist who listens attentively	2.91 (1.36)	2.91 (1.26)	**2.90 (1.26)**	3.01 (1.23)	.79			
7	Role play	3.34 (1.33)	3.72 (1.45)	3.41 (1.31)	3.96 (1.25)	.74			
9	Getting a personal coach	3.24 (1.33)	3.21 (1.35)	3.60 (1.39)	4.15 (1.50)	.71			
27	Seeing a therapist who requires you to face your fears	**2.72 (1.25)**	**2.87 (1.30)**	3.39 (1.34)	**2.83 (1.20)**	.67			
29	Seeing a therapist who you enjoy talking to	2.93 (1.50)	3.27 (1.42)	3.07 (1.30)	3.04 (1.32)	.66			
2	Talking about possible childhood causes	3.26 (1.41)	3.35 (1.51)	3.97 (1.62)	3.80 (1.38)	.65			
3	Practicing the fear-evoking activity	3.04 (1.55)	3.00 (1.43)	3.70 (1.38)	4.40 (1.52)	.57			
23	Imaging yourself doing the things you find stressful	4.00 (1.64)	4.05 (1.61)	4.11 (1.44)	3.98 (1.54)	.54			
11	Fear exposure with the assistance of individuals who do not share the fear	4.09 (1.69)	4.31 (1.66)	5.01 (1.43)	4.63 (1.51)		.83		
15	Indirect fear exposure	4.37 (1.56)	4.13 (1.51)	3.90 (1.41)	4.69 (1.46)		.83		
14	Actively practicing activities that are likely to evoke a fear response	4.32 (1.62)	4.05 (1.57)	4.13 (1.48)	4.23 (1.62)		.80		
13	Continuing with daily tasks regardless of anxiety	3.68 (1.39)	4.20 (1.38)	3.77 (1.41)	5.21 (1.29)		.75		
12	Behaviorally testing anxiety response e.g. in front of groups of people	4.07 (1.73)	3.96 (1.51)	4.03 (1.47)	4.42 (1.54)		.74		
4	Watching others engaging with the feared stimulus	3.84 (1.60)	4.33 (1.47)	4.20 (1.47)	4.96 (1.43)		.70		
16	Joining a leisure group that regularly engages in the feared activity/stimulus	2.77 (1.54)	4.33 (1.48)	5.00 (1.24)	4.43 (1.35)		.57		
21	Waiting to feel better	4.36 (1.51)	5.00 (1.43)	4.70 (1.47)	4.94 (1.58)			.82	
18	Avoiding anxiety provoking situations	**6.37 (1.29)**	5.41 (1.40)	4.93 (1.57)	5.39 (1.47)			.78	
19	Taking up a more carefully controlled lifestyle	5.62 (1.71)	3.86 (1.40)	4.98 (1.42)	5.16 (1.57)			.76	
30	Taking time off of work	5.64 (1.55)	**5.71 (1.43)**	5.00 (1.61)	5.81 (1.44)			.76	
22	Waiting a few years to get more mature	5.47 (1.52)	5.65 (1.32)	**5.64 (1.37)**	**6.05 (1.21)**			.70	
20	Taking medication	3.79 (1.52)	3.76 (1.48)	4.25 (1.39)	4.70 (1.54)			.62	
6	Reading self-help books	4.61 (1.31)	4.62 (1.26)	4.75 (1.32)	4.63 (1.30)				.73
24	Getting hypnosis	3.90 (1.64)	3.89 (1.59)	3.78 (1.41)	3.71 (1.62)				.63
5	Taking alternative medicine	5.23 (1.44)	5.17 (1.36)	5.32 (1.41)	5.23 (1.33)				.54

Note: These ratings are rated on a seven-point scale. The lower the score the more effective the therapy is thought to be for that disorder and vice versa.

*Abbreviations*: PTSD = Post-traumatic stress disorder; OCD = Obsessive compulsive disorder.

Although the treatments that were rated the least effective were more disorder-specific, efficacy patterns across conditions were apparent. Waiting a few years to get more mature (item 22) was rated the least effective treatment for PTSD (*X* = 5.64) and OCD (*X* = 6.05) and among the least effective in treating agoraphobia (*X* = 5.65). Avoiding anxiety provoking situations (item 18) and taking time off work until recovery (item 30) were both rated among the least effective in treating agoraphobia (*X* = 5.41; *X* = 5.71), OCD (*X* = 5.39; *X* = 5.81), and social phobia (*X* = 6.37; *X* = 5.64). Alternative medicine (item 5; *X* = 5.32) and watching films where similar trauma occurs (item 11; *X* = 5.01) received low efficacy ratings for PTSD and taking up more solitary hobbies (item 19; *X* = 5.62) was rated among the least effective in treating social phobia.

The 30 items (per problem) were then subjected to exploratory factor analysis (with VARIMAX rotation) to reduce the number of variables. Four factors emerged loading a total of 25 items, accounting for 69.35% of the variance. Factor loadings are shown in Table 1. The first factor was labeled *talking therapy treatments*, such as seeing a therapist and talking about possible childhood causes. It loaded nine items and accounted for 29.3% of the variance. The second factor loaded on the behavioral therapies, including activities that require the individual to face their fears. This factor was labeled *Fear confrontation therapies*, loaded seven items and accounted for 13.99% of the variance. The third factor was labeled *Fear avoidance therapies* because of its emphasis on removing one's self from potentially anxiety-provoking situations, including waiting to mature, taking time off work and taking medication. It loaded six items and accounted for 8.98% of the variance. The fourth factor was labeled *Alternative therapies*, such as using self-help books, hypnosis, and alternative medicines. It loaded three items and accounted for 5.11% of the variance.

Table 1 shows that, across disorders, participants rated talking therapies as more effective than fear confrontation and fear avoidance therapies as well as alternative therapy treatments. In addition, both fear confrontation therapies and alternative treatments were rated as significantly more effective than fear avoidance therapies. Taken together, talking therapies were generally rated the most effective and fear avoidance therapies the least effective across all four anxiety disorders.

A series of paired sample t-tests were performed to evaluate the perceived efficacy of each group of therapies on the four anxiety disorders. Talking therapies were rated significantly more effective in treating social phobia than agoraphobia (*X* = 3.27, SD = .92), PTSD (*X* = 3.44, SD = .87), and OCD (3.60, SD = .81). Talking therapies were also rated significantly more effective in treating agoraphobia and PTSD (*X* = 3.44, SD = .88) than OCD (*X* = 3.60, SD = .81).

Fear confrontation therapies were rated more effective in treating social phobia (*X* = 3.85, SD = 1.17) than agoraphobia (*X* = 4.17, SD = 1.03), PTSD (*X* = 4.30, SD = .95), and OCD (*X* = 4.66, SD = 1.13). When compared to the treatment of OCD, fear confrontation therapies were more effective in treating agoraphobia and PTSD. The efficacy ratings of fear confrontation therapies in treating agoraphobia were not found to significantly differ from those given for PTSD.

Fear avoidance therapies were rated as significantly more effective in the treatment of social phobia (*X* = 5.20, SD = .99), agoraphobia (*X* = 4.90, SD = .83), and PTSD (*X* = 4.92, SD = 1.07) than OCD (*X* = 5.38, SD = 1.08). When compared to the treatment of social phobia, efficacy ratings were also significantly higher for agoraphobia and PTSD. However, no significant differences were observed for the perceived efficacy of avoidance therapies in the treatment of agoraphobia and PTSD.

There were no significant differences between the efficacy ratings of alternative therapies in the treatment of social phobia (X = 4.58, SD = 1.11), agoraphobia (*X* = 4.56, SD = 1.07), PTSD (*X* = 4.62, SD = 1.04), or OCD (*X* = 4.52, SD = 1.12).

### Pain ratings of psychotherapy treatments

Table 2 shows the mean responses to each item (1 = very painful, 7 = Not at all painful). Results suggest a common pattern across disorders. Specifically, fear confrontation therapies were rated the most painful across treatments. These including taking up a lifestyle, wherein fear exposure is likely (item 11: social phobia *X* = 3.50; OCD *X* = 3.51; item 12: social phobia *X* = 2.72; agoraphobia *X* = 2.94; PTSD *X* = 3.33; OCD *X* = 3.15), discussing childhood causes (item 2: social phobia *X* = 3.57; PTSD *X* = 3.43; OCD *X* = 3.52) and practicing the fear-evoking activity (item 3: agoraphobia *X* = 3.51; PTSD *X* = 3.08). Therapies involving fear avoidance however, were rating among the least painful: Alternative medicine was rated the least painful across all four disorders (item 5: social phobia *X* = 6.10; agoraphobia *X* = 5.79; PTSD *X* = 5.86; OCD *X* = 5.70). Self-help books (item 6: social phobia *X* = 6.02; agoraphobia *X* = 5.66; PTSD *X* = 5.49) and taking up a more carefully controlled fear avoidant lifestyle (item 18: agoraphobia *X* = 5.48; OCD *X* = 5.56; item 19: social phobia *X* = 5.75; PTSD *X* = 5.69) were also rated among the least painful therapies. Together, results suggest that therapies involving graded exposure to the feared stimulus/situation are considered more painful than those involving minimal or no fear exposure.

**Table 2.** Means, standard deviations, and factor loadings for pain ratings of psychotherapy treatments.

			Anxiety	Disorder			Factor	Loading	
		Social				1	2	3	4
	Treatment	Phobia	Agoraphobia	PTSD	OCD	(41.68%)	(15.40%)	(6.87%)	(4.89%)
15	Indirect fear exposure	3.82 (1.87)	3.52 (2.05)	3.54 (1.92)	3.86 (1.91)	.89			
14	Actively practicing activities that are likely to evoke a fear response	4.40 (1.86)	3.70 (2.26)	3.85 (2.02)	3.75 (1.91)	.89			
11	Fear exposure with the assistance of individuals who do not share the fear	3.50 (1.93)	3.64 (1.89)	3.44 (1.91)	3.51 (1.93)	.87			
13	Continuing with daily tasks regardless of anxiety	3.72 (1.81)	4.18 (1.82)	4.00 (1.68)	3.65 (1.80)	.86			
12	Behaviorally testing anxiety response e.g. in front of groups of people	**2.72 (1.95)**	**2.94 (1.85)**	3.33 (1.87)	**3.15 (1.86)**	.77			
16	Joining a social group	4.15 (1.71)	4.13 (1.77)	4.03 (1.73)	3.92 (1.69)	.73			
3	Practicing fear confrontation	3.65 (1.89)	3.51 (1.84)	**3.08 (1.89)**	3.42 (1.85)	.70			
4	Observation	5.63 (1.61)	5.26 (1.51)	4.66 (1.84)	5.00 (1.71)	.67			
7	Role play	4.80 (1.61)	4.51 (1.70)	3.97 (1.86)	4.17 (1.70)	.62			
10	Attending a course	4.39 (1.93)	4.80 (1.83)	4.42 (1.72)	4.17 (1.88)	.60			
2	Talking about possible childhood causes	3.57 (1.56)	3.55 (1.66)	3.43 (1.72)	3.52 (1.64)	.50			
19	Taking up a more carefully controlled lifestyle	5.75 (1.52)	4.70 (1.94)	5.69 (1.44)	5.45 (1.58)		.82		
18	Avoiding anxiety provoking situations	5.45 (1.73)	5.48 (1.67)	5.47 (1.60)	5.56 (1.47)		.79		
22	Waiting a few years to get more mature	5.12 (1.85)	5.20 (1.75)	5.16 (1.81)	5.11 (1.76)		.76		
17	Learning to relax	5.47 (1.61)	5.15 (1.65)	5.49 (1.54)	5.24 (1.58)		.75		
30	Taking time off of work	4.97 (1.75)	5.13 (1.64)	5.04 (1.70)	5.07 (1.74)		.75		
21	Waiting to feel better	5.16 (1.51)	5.23 (1.55)	5.20 (1.53)	4.96 (1.54)		.75		
28	Seeing a therapist who listens attentively	4.70 (1.64)	4.86 (1.50)	4.60 (1.54)	4.70 (1.55)			.87	
29	Seeing a therapist who you enjoy talking to	5.20 (1.46)	5.10 (1.51)	4.96 (1.60)	4.98 (1.56)			.85	
26	Seeing a therapist who is supportive and sympathetic	4.81 (1.59)	4.70 (1.51)	4.60 (1.52)	4.71 (1.56)			.79	
8	Going on a training course	4.83 (1.55)	4.50 (1.58)	4.58 (1.51)	4.71 (1.56)			.65	
9	Getting a personal coach	4.87 (1.69)	4.52 (1.61)	4.58 (1.58)	4.62 (1.67)			.64	
1	Taking tranquilizers	5.58 (1.72)	5.18 (1.68)	5.21 (1.72)	5.14 (1.76)				.81
6	Reading self-help books	6.02 (1.26)	5.66 (1.41)	5.49 (1.42)	5.45 (1.43)				.56
20	Taking medication	5.45 (1.67)	5.43 (1.56)	5.46 (1.53)	5.55 (1.54)				.55
5	Taking alternative medicine	**6.10 (1.34)**	**5.79 (1.36)**	**5.86 (1.40)**	**5.70 (1.44)**				.55
24	Getting hypnosis	5.39 (1.50)	5.35 (1.47)	4.66 (1.84)	5.15 (1.58)				.50

Note: These ratings are rated on a seven-point scale. The lower the score the more painful the therapy is thought to be for that disorder and vice versa.

*Abbreviations*: PTSD = Post-traumatic stress disorder; OCD = Obsessive compulsive disorder.

The items were then subjected to exploratory factor analysis (with VARIMAX rotation). Each person made four ratings so there were 426 ‘subjects’ for analysis. Four factors emerged loading a total of 26 items, accounting for 72.87% of the variance. Factor loadings are shown in Table 1. The first factor loaded on the behavioral therapies including joining a leisure group and behaviorally testing the anxiety response and was labeled *High exposure*. It loaded eleven items and accounted for 29.3% of the variance. The second factor was labeled *Fear Avoidance* because of its emphasis on removing one's self from anxiety provoking situations. It loaded six items and accounted for 13.99% of the variance. The third factor was labeled *Gradual talking exposure* and it loaded items such as seeing a therapist or getting a personal coach. It loaded five items and accounted for 8.98% of the variance. The fourth factor was labeled *No exposure alternatives*, such as using self-help books, hypnosis, and alternative medicines. It loaded five items and accounted for 5.11% of the variance.

### Multiple regression analysis

A series of four regressions was then computed to examine the influence of demography (age, gender, marital status, and education) and knowledge of mental illness on the efficacy and pain ratings of psychotherapy treatments. Only the regression on the first factor proved significant (F(5, 73) = 2.46, *p* < 0.05; *R*^2^ = .14). It showed that younger subjects (*β* = 0.388, *t* = 2.62, *p* < 0.05) rated talking therapies (Factor 1), as more effective than older subjects.

The same set of regressions was computed to identify individual differences in the pain ratings of psychotherapies. However, results were non-significant for all four factors (*p* > 0.05), suggesting no significant individual difference predictors of lay perceptions concerning the psychophysical side-effects of psychotherapy treatments.

## Discussion

The decision to undergo psychotherapy and the experience of therapy once committed is presumably influenced by the individual's theories about, and expectations of, treatments and their outcomes. This study set out to contribute to the growing corpus of lay theories research on mental health literacy by investigating lay efficacy beliefs of various therapies, varying in fear exposure, for four relatively common anxiety disorders – social phobia, agoraphobia, PTSD, and OCD. It tested three hypotheses, two of which were supported.

The findings are broadly supportive of previous efforts, suggesting that the general public is able to differentiate between the efficacies of interventions for different anxiety disorders (Furnham et al., 1992). There seem to be consensually held optimistic and generally realistic conceptions about the usefulness of psychotherapy treatments and counseling that involve fear exposure, despite knowledge of the psychophysical side effects that these therapies often entail (Chen et al., 2000; Furnham & Wardley, 1990; Furnham et al., 1992; Jorm et al., 2000). However, the influence of sociodemographic variables and knowledge of mental illness, as predictors of this effect, was less consistent with that reported in previous research.

Supporting hypothesis 1, cognitive-based talking therapies were rated the most effective across the four anxiety disorders; but also the most painful. Talking therapies include seeing a therapist or personal coach, talking about possible childhood causes, and group therapies that often involve talking to someone who is trained to recognize negative affect and its influence on mood and behavior. Cognitive and behavioral therapies have been established as empirically supported treatments for anxiety disorders (e.g. Barlow, 2002; Chambless & Ollendick, 2001) that entail cognitive restructuring, anxiety management skills, and some form of exposure. While the structure and style of counseling programs vary between psychotherapists, counseling is usually considered most helpful in the treatment of psychological disorders (McKeen & Corrick, 1991). Of the talking therapies investigated, subjects rated seeing a therapist who requires clients to face their fears as the most effective psychotherapy treatment for three of the anxiety disorders tested (social phobia, agoraphobia, and OCD). Perceptions of a therapist's effectiveness stem in part from his or her ability to induce the discomfort required to mobilize client change and to be a trusted, accepting expert who can assist with this discomfort (Tonsley & Harris, 1976). Lay perceptions of the importance placed on the intensive social contact with a therapist mirror the findings of Furnham et al. (2001) in the treatment of depression.

Seeing a therapist whose treatment program requires the client to face gradual exposure to the feared stimulus/event either through imagination or in real time (i.e. graded exposure) is now one of the most utilized psychotherapy interventions and its success in treating anxiety disorders has been well-documented (e.g. Emmelkamp, 2003; Parsons & Rizzo, 2008). However, it does seem that many lay people appear to believe that ‘tea and sympathy’ is what works well, whereas the professionals stress the importance of ‘facing your fears’ and ‘performing appropriate’ actions to get better and cope well.

Interestingly, seeing a therapist who listens attentively was rated the most effective treatment for PTSD. The recurrence of intrusive memories and the feelings of helplessness and loss of control that often characterize PTSD require a therapist who can be receptive while the client relays the often fragmented, indelible images of the past. Moreover, on a conceptual level, the diverse traumatic events that can result in PTSD often require treatment to better understand individual contextual issues including the ethno-cultural and societal aspects of the traumatic event (Moodley & West, 2005). Thus, perceived effective psychotherapy for PTSD requires case-specific sensitivity to contextual information via the therapist listening to self-reported recurring memories of the client.

Supporting hypothesis 2, fear avoidance therapies were rated the least effective and alternative medicine was rated the least painful across all four anxiety disorders. Specifically, waiting a few years to ‘get more mature’ was rated the least effective for PTSD and OCD and avoiding social situations was rated the least effective for social phobia and taking time off work was considered the least effective for agoraphobia. These findings do not lend support to some previous studies that have suggested cure specific perceptions of the efficacy of different cures (Furnham & Henley, 1988; Henley & Furnham, 1988; Knapp & Karabenick, 1985). It is of course possible that subjects were not responding to the perceived efficacy of treatments but rather to their popularity or exposure among lay people. Equally in this study all the disorders were anxiety disorders, whereas previous studies have had a much wider range of disorders.

The efficacy of individual therapies appears to be generalizable across anxiety disorders (Furnham, 2010). That is, individuals believe that (based on the descriptions offered in the questionnaire) cognitive and psychodynamic therapies are useful and behavioral and physical therapies as less useful in treating all anxiety problems. Meta-analytic findings clearly support the efficacy of combined cognitive-behavioral treatments for anxiety disorders (see Deacon & Abramowitz, 2004 for a review). That considered, therapists should be managing naivety and false optimism about the effectiveness of psychotherapy interventions at the beginning of client interactions to encourage client investment and adherence to the treatment program.

Taken together, in line with the series of findings based on adults, students and therapists by Furnham and Wardley (1990, 1991; Furnham et al., 1992), subjects perceived cognitive and psychodynamic therapies as most effective for a range of anxiety disorders while the behavioral and physical therapies were rated least effective. This certainly seems the opposite of much common practice.

However, one reason for these results may be to do with the inaccurate and unreliable wording of the items. Thus, it could be argued that items 11 and 12 do not sufficiently represent current practice in behavior therapy. Further, some items (e.g. 14 and 15) fail to capture the empathic nature of the therapy (Blackburn, James, Milne, & Reichelt, 2001). Indeed, it could be argued that item 12 is rated both painful and inefficacious because it conjures up an aversive scenario of a group of random people witnessing painful exposure with no sense of group support. All therapies attempt to get people to see themselves in common situations but give help and support to cope with them, and group therapy does it supported by others.

In terms of differential demographic correlates of efficacy and pain ratings of psychotherapy, subject age was the only significant predictor of the perceived efficacy of cognitive-based talking therapies. Younger subjects were more optimistic about the efficacy (i.e. usefulness) of talking therapies in the treatment of anxiety disorders than their older counterparts. This finding lends support to previous research that has identified older people to be generally more pessimistic about the potential benefits of therapy and as less likely to utilize mental health services (Furnham & Wardley, 1990; Leong & Zachar, 1999; Wong, 1994).

Current society can appear particularly avoidant of confronting negative aversive affect, resorting to chemical ‘fixes’ (alcohol, prescribed and illegal drugs) instead, so it is both interesting and heartening that younger people see the therapeutic value in facing their emotional issues.

The finding that knowledge of mental illness was not significantly related to subject efficacy ratings failed to support hypothesis 3 and refutes previous research that has found a significant effect of knowledge and experience of psychology on lay perceptions of the efficacy of various psychotherapy treatments in the treatment of psychological disorders (Furnham & Wardley, 1991; Furnham et al., 1992). Thus, H_3_ was rejected.

There are two possible explanations for this finding. First, while knowledge and experience of mental illness has been found to be predictive of attitudes towards psychotherapy, previous research efforts have generally found very little variance in lay attitude scores is accounted for by individual difference factors (e.g. Furnham, 2010; Furnham & Wardley, 1990). Therefore, the decision to embark on psychotherapy may not be determined by factors relating to the individual such as socioeconomic status, education, and knowledge (Fischer & Turner, 1970) but rather factors relating to the availability, quality, and costs of the therapy. Alternatively, this finding could be attributed to the methodological design of the study. Findings are based on a relatively small sample, the majority of whom were based in the south-east of the UK, wherein the provision of psychotherapy and experience of mental illness is more likely than in other parts of the UK. Therefore, this finding could be the result of restricted variance in the knowledge/experience of mental illness scores.

All studies have limitations and this study is no exception. First, arguably, the findings are based on a small sample of better educated, younger lay people. They were clearly not representative of the population as a whole, and because some had some knowledge of psychology it is disputable as to whether they should be termed as ‘lay people’. Future studies would do well to use a larger and broader sample, making the generalization of findings to other settings and groups more viable. It would also mean that confidence in the factor analytic results would be stronger Secondly, no effort was made to enquire about participants’ direct exposure to the anxiety disorders investigated. Given their relatively common occurrence in the general population, it was assumed that subjects would be able to form some implicit theories regarding their cause and treatment based on direct experience. In order to extend the robust findings of Furnham et al. (1992), it would have been desirable to collect efficacy and pain ratings from professional psychotherapists for comparative purposes. Therapists tend to be selective, believing that therapies are suitable for specific psychological problems while lay people show generalized optimism, believing that therapies are suitable for a wide range of psychological problems (Furnham et al., 1992). Finally, some of the descriptions of the therapies may have been misleading and it would be desirable in further studies to have a more detailed and veridical description of the therapies so that participants could make a more informed judgment.
